# Lived Experiences of Educational Leaders in Iranian Medical Education System: A Qualitative Study

**DOI:** 10.5539/gjhs.v8n7p251

**Published:** 2015-12-16

**Authors:** Zohreh Sohrabi, Masoomeh Kheirkhah, Zohreh Vanaki, Kamran Soltani Arabshahi, Mohammad Mahdi Farshad, Fatemeh Farshad, Mansoureh Ashgale Farahani

**Affiliations:** 1 School of Medicine, Iran University of Medical Sciences, Tehran, Iran; 2 School of Nursing, Tarbiat Modarres University, Tehran, Iran; 3 School of Medicine, Iran University of Medical Sciences, Tehran, Iran; 4 School of Dentistry, Tehran University of Medical Sciences, Tehran, Iran; 5 School of Nursing & Midwifery, Iran University of Medical Sciences, Tehran, Iran

**Keywords:** education quality, education managers, medical education, qualitative study

## Abstract

**Introduction:**

High quality educational systems are necessary for sustainable development and responding to the needs of society. In the recent decades, concerns have increased on the quality of education and competency of graduates. Since graduates of medical education are directly involved with the health of society, the quality of this system is of high importance. Investigation in the lived experience of educational leaders in the medical education systems can help to promote its quality. The present research examines this issue in Iran.

**Methodology:**

The study was done using content-analysis qualitative approach and semi-structured interviews. The participants included 26 authorities including university chancellors and vice-chancellors, ministry heads and deputies, deans of medical and basic sciences departments, education expert, graduates, and students of medical fields. Sampling was done using purposive snowball method. Data were analyzed using conventional content analysis.

**Findings:**

Five main categories and 14 sub-categories were extracted from data analysis including: quantity-orientation, ambiguity in the trainings, unsuitable educational environment, personalization of the educational management, and ineffective interpersonal relationship. The final theme was identified as “Education in shadow”.

**Conclusion:**

Personalization and inclusion of personal preferences in management styles, lack of suitable grounds, ambiguity in the structure and process of education has pushed medical education toward shadows and it is not the first priority; this can lead to incompetency of medical science graduates.

## 1. Introduction

Proposing the crisis of quality in education especially in higher education has been associated with some movements to secure the quality of education in higher levels (Farokhnezhad & Peerdadiean, 2011). Improvement of higher education programs are the most important concerns of educational centers (Enayati & Zamani, 2013). The quality of education is the key to achieve and maintain success. It is defined as a collection of suitable features which are realized through competent professors, related educational content, active learning techniques and motivation of the students (EFA Global Monitoring Report, 2005). Medical education is a part of higher education which is directly involved with the lives of people and the health of society depends on the quality of services provided by health system. Empowered medical graduates have an important role in providing high quality services (Fasehi Harandi & Soltani Arabshahi, 2004). There is effort running and promoting the quality of education (Rahimi, Zarooj Hosseini, & Darabian, 2012).

Iran medical education system has combined education with the service-provision. This is proposed as a new unique paradigm in the world. In Iran, Medical universities are the authority of education and provision of medical services in actual life (Integration of Staff Development Center of the Iran Ministry of Health and Medical Education, 2008). Development of medical education systems in a purposeful, health-oriented, just, and responsive way which meets the needs of society is being worked on; and training efficient, skilled, and committed human forces with professional moral based on the geographical needs of the country are at the agenda for the leaders of this field (Health policy document in the Islamic Republic of Iran, 2015). Evidence showed that the positive effect of medical system training in reducing the mortality and morbidity rates in the society (Tokuda et al., 2010). Medical system authorities and programmers try to produce graduates who are able to save the lives of patients and promote the health of society. Social accountability is one of the main elements of management of health and care system (Ross & Mirowsky, 2010). Educational leaders are responsible for maintaining and guaranteeing the quality of education (Larijani, 2015). Unfortunately, despite the attempts done, about 90% of medical students evaluate the quality of education as medium (Iran Manesh et al., 2014) and there is a distance among the perception of students from the present quality of education and the educational standards (Larijani, 2015). The education experts also report a decline of quality in medical education of Iran (Ahmady, 2015). Inefficiency of medical graduates brings about stress and mental tension for them (Gibbs & Coffy, 2004), and it endangers the health of society and incurs high financial costs due to unnecessary para-clinical and pharmaceutical demands to the society and government; these issues emphasize more on the necessity of explaining the effective factors on the quality of medical education. 30 years has been passed since the merger of medical education and service provision sections in Iran but the studies in this field are still few. Explaining the lived experience of educational leaders regarding the quality of education in medical system of Iran can help the programmers and educational officials in promotion of its quality.

## 2. Methods

In this study the descriptive method of content analysis was used. When the goal is analyze a phenomenon, content analysis is used. One of the three approaches used in qualitative analysis of data is the common way in which coding and categorization of raw data are direct using an inductive approach (Hsieh & Shannon, 2012). The goal of present research is to describe the phenomenon of education quality in medical education system of Iran. Content analysis of educational leaders’ experiences lead to its description and identification of the facilitative and disincentive factors. After obtaining the necessary permissions from ethical committee and letter of introduction from graduate department of Iran Medical University, sampling was done using purposive snowball method. The researcher explained the goals of research and obtained the permission of participants to be studied and interviewed. They were informed that after admission, they can leave the study in any stage. They were assured of the confidentiality of their personal information (name, last name, etc.) in the reports. Interviews were first without structure and then became semi-structured. In this study, the interviewees consisted of three Ministry officials in the domain of educational programming of general medicine course, the manager of medical center of development and research, three university heads of medical universities, board members and the chairman of medical research center, 1 university vice-chancellor of education and group manager, 4 deans of medical science faculties and board members of specialized fields, 2 education vice-chancellors of faculty, 2 research vice-chancellors of faculty and department, 1 manager of graduate studies office, 3 managers of educational groups, 1 faculty member and the responsible of educational programming of theory and clinical lessons of department, 1 graduate of medical education and 3 students of medical sciences. The interview time was about 20 to 110 minutes. Interviews were started with questions regarding the role and function of interviewee and recorded with and MP3 player. The interviews were listened to several times and transcribed in word2007, analyzed and coded.

In qualitative content analysis, the main issue is deciding about two things: first, the unit of analysis must be defined and decide the analysis is focused on manifest content or latent content (Hsieh & Shannon, 2012). Manifest content refers to the analysis of what the text says, it is observable, clear and distinct, and usually appears in the categories. The latent content refers to the deep meaning of content and represents itself in the themes (Graneheim & Lundman, 2004). In this study the whole interviews were defined as the unit of analysis. Sentences and/or paragraphs were considered as the units of meaning. These units were summarized in terms of content and elements, compared, and conceptualized at abstract level based on their latent concept, and were named by codes. Codes were compared with each other in terms of similarities and differences and categorized under more abstract labels. By comparing the categories with each other, and deep contemplation the latent content of the data was introduced as the theme of study.

The accuracy and strength of research was investigated using the proposed criteria by Guba and Lincoln. The researcher tried to increase the credibility of research through long engagement, participation, and interaction with the interviewees, as well as collecting valid data, doing member check and verification of data. Data collection and analysis was done under the supervision of research professors and experts to enhance the dependability of data. To increase the confirm ability of data, the ideas of university professors and their review was used. For the transferability of study, it was tried to present a rich description of research reports for the evaluation and application of research.

## 3. Findings

The participants of this study included both male and female managers, faculty members, education experts and programmers at the level of ministry, university, and department-with 3-33 years of work experiences and academic ranks such as professor, associate professor, assistant professor, and lecturer. Moreover, in order to find deep information regarding the quality of medical education system, some interviews were done with students and graduates of general medicine, specialization, and fellowship. An educational focus group was used to reach a deeper understanding of education phenomenon. The primary codes were extracted; after several reviews, they were summarized and categorized based on similarities and differences. They were determined in the form of one theme, five categories, and 14 subcategories and coded conceptually and abstractly based on their nature.

The categories included: quantity-orientation, ambiguity in the education, ineffective interpersonal communication, unsuitable educational environment, personalization of selecting education management. They are presented in the following table.

**Table 1:** The extracted themes from the experience of participants

Theme	categories	Subcategories
Shadowed education	Quantity-oriented	•Focus on academic degree and specialization •Commercialization of education
	Ambiguity in the education	•Ambiguity in education structure •Ambiguity in curriculum and its management •Ambiguity in educational program and jobs •Ambiguity in the responsibilities of jobs •Ambiguity in feedback, evaluation, and job promotion of professors
	Unsuitable educational environment	•Agonizing educational environment •Mismatch between educational background and future job’s description •Lack of regular supervision and educational feedback •Lack of educational levels
	Personalization of Educational Administration	•Instability and ambiguity in the selection and election of management
	Inefficient interpersonal communication	•Inefficient communication between lecturers and students •Inefficient communication between lecturers and educational leaders

The main theme of this study is “shadowed education” which refers to the marginalization of education in the medical system. Some of the participants indicate that “now in the ministry of health and treatment, medical education is in the shadow, inside the system itself; as a teacher, a trainer, my concern should be teaching in order to train a skillful nurse, a good midwife or an excellent doctor; but the system expects me to be involved in treatment, to produce articles and papers and science. I have to do research (No. 6)”. The problem is that we were responsible for education but we did not pay the attention it deserves to the education. Research section says you have to have published articles, treatment says I pay for these and education says I pay your salary and you do the research and treatments; what do you expect from education?” (No. 13). Participant No. 7 says about the priorities of educational system: “the first priority is treatment, second research, and education is the last. The first priority is treatment because there is money and insurance, and public complaint; research is backed by credit and articles; it is attractive for me as a faculty member…what about education? It is boring and exhausting; it has no benefit for me as a teacher”. Participant No. 2 says in the Medical University the faculty members try to promote themselves by writing papers, they research and publish ISI articles to climb the ladders. Those who are not interested in academic ranks or don’t have its taste try to increase their income; they leave education behind. They treat patients who have high tariffs such as diagnostic and invasive procedures so that they encounter less complaint, and pass the less serious patients to their interns and assistants and keep their charge. Education in this situation in this system is merger of third-class citizen”.

One of the effective factors on “shadowed education” is focusing on quantity. Rapid development of universities and quantity has damaged the process of evolution and promotion of higher education in the country. Participant No. 2 says: “the merged education system was set in Iran in order to increase the number of medical colleges in this ground very fast and train the required human resource in the field of health and replace them with medicines that were paid in dollars. We quickly had a suitable number of graduates of medical and paramedical faculties that we had just established, and this relieved us from paying costs in dollar for medical services”. The head of a medical university states that: “we don’t want many students now to have mass production, we should accept a few, select them, and train them well” (No. 7).

One of the subcategories of quantity-orientation is the attention to academic degrees and specializations. Participant No. 6 says: “despite the considerable growth in the quantity of medical sciences, the education in Bachelor’s, Master’s, MPH, PHD, DMS, all focus on the treatment”. Participant No. 4 says: in the last 3-4 years the growth of students’ population, the clinical student has been totally marginalized. We were not medical, our PhD students has reached from 2-3 students to 12-14 per year, what are they going to do with 20-30 masters student? Get along with each other, until it is finished. In medical section residents are increased, the hospital is full of students like a dormitory”.

Commercialization of education with focus on the treatment dimension is another subcategory of the second category. Participant No. 6 says now the focus is on the treatment, health and health criteria are abandoned. My experience as a common man, as a student, as a faculty member, a public servant and a manger of health system shows that treatment is not going the right way.” Participant No. 4 says: “in general, what is not important at all is quality, they either want to involve the youth, or make money. This international and evening courses are just about money, we put the 1^st^ and 40000^th^ student in one class, and say we don’t have building, we have gathering, it is a gathering education. The name of our medical education is gathering education”. Medical students face commercialization every day: “a professor that prescribes too many medicines; we ask why? He says a part of our job is business. The patient says: do I have an injection? He says: here you are. He writes whatever the patient asks to keep the patient. Anyone with any level of education falls in this path and asks for extra medicine and experiments for the patent. Participant No. 21 says: resident visits a patient and receives his money, while the attending physician should visit the patient. The benefits of attendant physicians in the hands of resident, they have direct working relationship. Resident tries to never upset the attendant. Resident training is routine and general medicine training is not any better”.

The next category is ambiguity in education and the subcategory of ambiguity in educational structure. “Absolutely there is no comprehensive educational system, there is no will. Those who present the education, only provide education, there is no infrastructure. For example Ms. X holds X workshop, it is all about duty. To say that something has been done, and some workshop has been held, we took the credit. It is not important at all how much it affects the system (N. 20). Participant No 4 says: “the structure of our education is problematic, merging it with the service section made it so service-oriented that it is not clear whether it is training or treatment. They say autonomous educational hospital; this is a paradox, completely paradoxical. Autonomous educational hospital with payment system means as the case is less complicated the physician earns more, if the hospital wants to spend on training with complicated cases it will go bankrupt. It is indeed bankrupt already without such cases; with education it is definitely bankrupt”.

Ambiguity in the educational curriculum and its management is another subcategory of ambiguity in education. Participant No. 28 says “we give the educational syllabus in the ministry. Ministry has no authority on the program and the universities decide. We have not a curriculum committee, every one decides personally”. Participant No. 1 says “ministry writes the curriculum, then communicates it to the faculty, then send it to the group; or there is board members in the group, the views of board is practiced or they work according to their personal views and write the lesson plans. There is no supervision. In clinical lessons the issue of absence and presence is taken seriously but there is no administrative plan”. No. 21 says “now the basic procedure for interns is not clear; that is those procedures that general physicians should learn and go is not clear”. One of the interns says: “we give the basic prescription to all patients, and then the physician comes and changes it. When we ask the reason he says medicines are changed, it is not useful for you. These things happen because of unclear objectives. Before a student can leave, they must say how many patients should he visit and know the treatment, important points about them but we don’t have such a thing”. Participant No. 1 says “the process of curriculum administration of the university should be monitored. They plan it and teacher understands it in a way and the students understand it in another way. We don’t have any supervision; it needs a good preceptor and mentoring”. Supervision and monitoring of educational curriculum is one of the effective strategies for removing ambiguities.

The next subcategory is ambiguity in the educational program and job tasks. “Our general medicine curriculum has not been revised for 30 years; we have fluctuations in the strategies. In curriculum review it should be crystal clear what we are going to train, family or general practitioner? (No. 15) “. One of the officials of general medicine section of Ministry of Health states that “we take 4200 new students every year; we must know that are they going to become family or general practitioners seven years later? More than half or a third of them will not be absorbed into family practitioner section. Does this field has the power to train every student as a family physician or is there the necessity to do this? (No. 18)

Among the other ambiguities of education is ambiguity in the functions of different job areas. No 21 says “job functions must be clear. It should be clear what an intern should do? A midwife should do? Or a woman’s specialist? From one hand, we train woman’s specialist, on the other hand a midwife bachelor, masters, or doctorate…the job description is not clear. Whether a specialist should take the natural birth degree? Can she? Does it have an effective expense? We have ambiguities in the field of job functions and responsibilities.”

Among the other ambiguities is ambiguity in job promotion of educational professors. Educational professors promote based on the number of published papers and ISI articles. No. 23 says “now the evaluation of professors is more based on the research, how many ISI articles they have. We make rules and just watch how many articles you have. We are all following the contemporary world. When they say they are valued when they have research and published papers, they move toward research. This is my personal experience; this is why the education system has damaged like this”. No. 24 says “to promote my rank I gave my degrees and documents, they just looked for my PubMed and ISI articles. My teaching experience and administrative activities were not seen, the value of faculty member is related to the number of his articles not the quality of his mentoring. Nothing is more important than articles”. The strategy of encouraging educational faculty members is not appropriate and educational criteria are not considered in there. No. 2 says: more value should be paid to educational powers in promotion criteria, or at least consider it as an equivalent of research so that the applicants tend to education. The promotion criteria should be revised to absorb more attention to it”.

The next ambiguity is ambiguity in feedback and teachers’ evaluation. No. 14 says “I use a method for training, teaching, and taking exams because no one gives feedback, no one says my job is good or bad, I continue the same method and tell my students that if you want to be successful keep this method; while I’m not sure about it. This means that we are training our next generation with the methods that we don’t know whether they are correct or not”. Another participant says “evaluation is good when a change is made. When nothing changes, what’s the use of it? They should not waste our time. When they evaluate nothing changes. When there is one person at this position, and he is the only one what is the evaluation for? (No. 19). participant No. 22 says if a professor has no lesson plan and does not teach based on standards, the development center can say this lecturer must be banished from teaching for one semester to compensate and comeback. But when it does not have the power, and when it says to the medical university that his training is very low quality and hears that we have no other lecturer, we need him. What is an evaluation for? (No. 22).

Practically, suitable feedback is not given to educational professors based on the evaluations. Their promotion is based on other criteria such as research papers.

Unsuitable educational environment is the next category. The agonizing educational environment is another subcategory. Participant No. 26, a graduate of odontology says “now that I’m soldier, there is better than the diagnosis division of department. They are better than the X doctor of diagnosis division”. No. 24 says” training is agonizing, so agonizing that I heard students say can we get rid of this hell and become a doctor before we die”. This statement reveals the necessity of revision and improvement in the educational environments.

The next subcategory is mismatch of educational background and the future job description. One of the participants says “Hospital is not the instance of a future work place of the medical student. We worked in our education with schizophrenic and bipolar patients who constitute less than 10 percent of our real patients in the future. we have been well-trained for 4 years, we did not see any education for hospitalization of patients and professional needs, for example we faced a disloyalty case that we were not trained for it” (participant 3). Participant No. 21 says “what is the necessity for an intern to work in respiratory specialized clinic? Is he supposed to work later in that section? No. interns should be educated in the general section. They are educated in sections that are not useful when they work out there; there is no need to such an education”.

Lack of supervision and regular feedback in the education is another subcategory. One of the participants says “we appealed that check the absence of students. We have such cases that a student was studying medicine in another city and no one has checked the absence of student, the education department of university has found this and told us” No. 12 and Participant No. 15 says “our weakness is in control and supervision. I don’t mean spying. Do we really know that what is happening in our clinical system? Unfortunately we had a clinical professor that has attended two days instead of 9 days to the hospital and gave all students 19-20 to mute students. Even sometimes the authorities know, but they are indifferent and don’t make a difference between punctual me and the disordered person. The main education is done on the patient and the supervisor professor. But these are not the problems now. The attending physician of emergency works with resident of emergency. The attending physician of surgery works with resident of surgery in the emergency. God knows only about intern and extern” (No. 21). Participant No. 18 One of the general medicine authorities of ministry of health, education and treatment says that “curriculum is given to the groups. Every unit is given to a lecturer. If there are several professors each of them does his work. There is no supervision unless a student complains. We don’t have day to day care and supervision”.

One of the factors of shadowing the education is lack of regular supervision. No. 26 the manager of development center of medical education says “the education and service systems are intertwined. As a faculty member, I have to act as a lecturer and mentor and also a health provider. In such a situation, neither the Treatment nor the Education must say I should visit patients in the educational hospital and the resident monitors. My resident works and learns, nowhere is like this. It has a solution is to provide feedback and supervision”. Interns and stagers are not under direct supervision of professors and they are abandoned in the hospitals and used as cheap labor force. Their educational and evaluation is done by residents who do not have enough experience and knowledge of education, supervision and control. General medicine students are not provided directly with the model professors. Lack of ranking and classification of education is another subcategory. “(No. 22).

No. 6 The group manager and head of an educational medical center says “Students, interns, and assistants are at three different levels. I separated the general medicine training from specialized training as the head of education department. General medicine training was always affected by residency training and it’s practically ignored. In many places it is being removed, by fellowship courses of training residents and stagers, while their educational needs are different with each other and the stager is damaged; because usually the majority of discussions tend to upper levels. The solution which my suggested it at many places is to separate general medicine training from specialized training”. Classification of education is one of the effective solutions in promotion of quality of medical education which is not done in many educational centers.

The next category is personalization of educational management. “In our education and treatment system, once they worked on clinical governance and then accreditation came and eliminated the former completely. We devoted so much time to clinical governance programs and they were all removed. This demotivates people. When I see in this collection that my time for 10-12 sessions of clinical governance was taken but it was never administered. We see no change in the system and the next management was not concerned with continuing it. The programs are not processed and implemented; they all depend on the idea of the responsible officials (No. 14). One of the interns said “The score is given based on the idea of lecturer. We had a student who has failed in theory and got a 19 score in the clinical section. Or the top student of theory classes who got a 12-13 at the clinical (No. 19).

Lack of stability of management and ambiguity in election and selection of educational managers is another important subcategory which affects the process of education. No. 14 says “in the system, one says I’m a manager now and I will be for three years, why I should make enemies to myself. This is why he doesn’t go for administration of plans. The most important involvement of mangers is that they don’t know how long they remain as a manager “The weakness of our system is that we select someone who has no experience, it happened in our university that someone who has just entered the university was selected as the educational deputy. In successful systems, first one becomes a group manager; when they proved their ability they may be selected as dean of faculty, then vice president and finally deputy of education of the university. There is a weakness in our programs we are walking on ice. We cannot make a strong move in the administrative grounds and implement our programs correctly.

No. 7 says “There is no consistency and stability in the decisions. The educational leadership suffers from imbalance and lack of uniform policy in macro levels. Now they say qualitative development. How we can say so much words in these 7-8 months then take our words back. Lack of managerial consistency and lack of stability in the decisions of mangers are effective factors on reduced quality of education.

Effective interpersonal communication is the next category. It includes two subcategories of inefficient relationship between professors and students, and inefficient relationship between professors and educational leaders. Participant No. 3 says “I try to establish a humanistic relationship rather than a formal one. Such a relationship is a priority in my job because I train masters and doctorate students the relations are more natural and humanistic”. This look is based on educational leadership but the students have a different narration of it. Participant No. 19 “The professors have nothing to do with the students where a resident is present. What we do is reorder and taking the patient to CT-scan and MRI and visiting new patients. We do this and they don’t want anything more of us. Very politely our work is finished”. Participant No. 22 student of medicine says “If we are engaged in the patient’s visit and diagnosis, we do all the other works. We take the blood to the lab, we inject, we fill in the record sheet…but in practice we have no part in the first decision making and diagnosis of disease. We are the cheap labor force. In many cases of medical education, the communication among professors, students, and colleagues is problematic or does not exist.

## 4. Discussion and Conclusion

Explaining the lived experience of educational managers regarding the quality of medical education system of Iran showed that despite the existence of a future perspective, the administrative plans are not in accordance with the ideal objectives and many ambiguities exist in the programs, administrative processes, educational structure, educational curriculum, supervision, and feedback. The study of Lee et al. (2008) also referred to some ambiguities in the goals of educational system and evaluation goals (Lee, Altschuld, & Hung, 2008), which is consistent with the present findings.

The educational and evaluative processes and structures in the medical education system of Iran are full of ambiguities; according to the ideas of education authorities we cannot expect a high quality of education from this system. In a successful educational institute the limit of authorities, responsibilities, policies, regulations, and strategic or operational programs are clear and at the hands of beneficiaries. The results of Bikmoradi et al. (2010) study on the challenges of scientific management in the medical universities showed that scientific management in medical universities is very inefficient and the majority of components of management structure are uncertain and ambiguous (Bikmoradi et al., 2010).

Khodaveisi et al. (2011) state that constant scientific evaluation of students, attention to spiritual and behavioral dimensions especially professionalization process are requisites of medical science (Khodaveisi, 2011). While the results of the present study introduce the effective factors as the lack of educational classification, lack of grounds and mismatch between educational ground and future job description, lack of supervision and regular educational feedback, focus on degrees and academic rankings instead of expertise and skill-learning. The issue of ambiguity in the monitoring and control leads to lack of regular monitoring of system, chaos in the educational system, reduced motivation and job satisfaction among professors, reduced quality of education, and declined scientific level of students. Paying attention to quantity, degrees, and specializations has affected general physicians’ education and students seek for having high-income fellowships. There is no coordination between their educational background and their future job functions. Usually the graduates pass their term of service in deprived rural areas while they receive their main training in the specialized educational medical centers. In rural areas, medical and diagnosis equipment and devices do not exist and it will not help to their future job functions. In this regard, similar results were found in China medical education and the researchers suggested that for promotion of general medicine education the training of health service providers is done in rural areas and more emphasis is made on general practice lessons than specialized ones. The studies show that at the beginning students are interested but gradually they become pessimist, intimidated, hopeless and depressed which can be explained by misbehavior with the students (Maida, Vasquez, & Herskovic, 2003). 68.5% of medicine students (6^th^ year) experienced misbehavior in the university environment including physical and verbal. Nagata-Kobayashi state that 81.6% of the female medical students and 61.2% male students have reported at least one case of verbal or physical misbehavior during their education (Nagata-Kobayashi etal, 2006). This is a very effective factor on demotivation of students and the effect of its deficient cycle shows itself in the relationship with patients, medical staff, and society. The results of present study indicate lack of efficient relationship among professor and students which will result in training passive, obedient, and subservient students. Lack of supervision and effective communication will prevent growth of talents, innovation and creativity in the students and makes them suitable for mental damages and disorders, depression, and internal complex and provides the ground for repletion of defective cycle of mental damages in the society.

Furthermore, the study showed that interns and stagers are not under direct supervision of professors. They are abandoned in the hospital and are used as cheap labor forces. Their education and evaluation is done by residents who don’t have enough knowledge and experience about teaching, monitoring, etc. They don’t participate actively in the treatment team. Such issues may have adverse effects on the development of medical argument, the quality of provided service to the patients, education and learning.

Low interaction between professors and students and lack of a role modeling function and teacher-student relationship is among the factors which affect the quality of education and treatment of patient (Tsouroufli & Payne, 2008). Lack of support and regular day to day supervision leads to lower preparation and powers of students (Zhang & Wildemuth, 2009). It also has a significant impact on efficiency and professional competency of students in providing service to the public. Poorghaneh (2014) conducted a qualitative study titled as the experiences of nursing students from clinical training. She refers to lack of proper relationship between members of healthcare team with the students, mentors, and personnel. This has a very great effect on the team-work and communication in educational medical centers (Poorghaneh, 2014).

Daily supervision of leaners, changing the curriculum of medical fields as a combination of medical science and humanities, with merging clinical skills and professionalism, changing the methods of learning and teaching from teacher-oriented to learner-oriented in a way that the process of learning becomes a lifetime activity and increases the ability of innovative and creative thought, classification of educational levels and categorization of faculty members to education, research, medical, administrative groups, promotion of the ranks based on the needs and functions of the related area and paying enough salary suitable with the provided service will be very effective on the promotion of medical sciences as well.

## References

[ref1] Ahmady S, Akbari Lakeh M (2015). Exploring the practical themes for medical education social accountability in Iran. Gastroenterology and Hepatology from Bed to Bench.

[ref2] Bikmoradi A, Mats B, Alireza S, Davoud K.-Z, Italo M (2010). Identifying challenges for academic leadership in medical universities in Iran. Medical Education.

[ref3] EFA Global Monitoring Report (2005). Understanding Education Quality.

[ref4] Enayati T, Zameni F, Nasirpoor D (2013). Assessing the quality of educational service in Mazandaran University of Medical Sciences using Seroquel Model. Journal of Health Promotion Management.

[ref5] Farokhnezhad K. H. N, Peerdadiean M (2011). Quality assurance of the quality of teaching and learning in the university system, the fifth Conference in monitoring the quality of the university.

[ref6] Fasehi Harandi T, Soltani Arabshahi S. K, Tahami S. A, Mohammad Alizadeh S (2004). Quality clinical training of the students of Iran University of Medical Sciences. Qazvin University of Medical Sciences Journal.

[ref7] Gibbs G, Coffey M (2004). The impact of training of university teachers on their teaching skills, their approach to teaching and the approach to learning of their students. Act Learn High Educ.

[ref8] Graneheim U. H, Lundman B (2004). Qualitative content analysis in nursing research: Concepts, procedures and measures to achieve trustworthiness. Nurse Educ Today.

[ref9] Hsieh H. F, Shannon S. E (2012). Three Approaches to Qualitative Content Analysis.

[ref10] (2008). Integration of Staff Development Center, Medical Education Department of the Ministry of Health and Medical Education, education, philosophy and process of implementing the integration of medical education in the Islamic Republic of Iran, in line with the provision of services to meet the needs of.

[ref11] Iran Manesh F, Hamza Moghadam A, Shafa M. A, Mohamadi E (2014). Evaluated the quality of education from the perspective of Neurology Medical Students steps Development of Medical Education. Journal of Medical Education Development Center.

[ref12] Khodaveisi M, Pazargadi M, Yaghmaei F, Alavi Majd H (2011). Evaluation of nursing education: A qualitative study. Journal of School of Nursing and Midwifery Beheshti University of Medical Sciences.

[ref13] Larijani B (2015). Medical education seminar on challenges. Academy of the Islamic Republic of Iran.

[ref14] Lee Y. F, Altschuld J. W, Hung H. L (2008). Practices and challenges in educational program evaluation in the Asia-Pacific region: Results of a Delphi study. Evaluation and Program Planning.

[ref15] Maida A. M, Vásquez A, Herskovic V, Calderón J. L, Jacard M, Pereira A, Widdel L (2003). A report on student abuse during medical training. Med Teacher.

[ref16] Nagata-Kobayashi S, Koyama H, Yamamoto W, Goto E, Fukushima O, Ino T, Koizumi S (2006). Student Abuse during Clinical Clerkships in Japan 2006. J Gen Intern Med.

[ref17] Poorghaneh C (2014). Nursing students'experiences of clinical education: A qualitative study. Holistic Nursing and Midwifery.

[ref18] Rahimi M, Zarooj Hosseini R, Darabian M (2012). Teacher evaluation by students: A comprehensive approach. Strides in Development of Medical Education.

[ref19] Rogstad K, Talbot M (2001). A preliminary study comparing attitudes of trainee doctors and their mentors, to compulsory educational supervision in postgraduate medicine. Mentoring and Tutoring.

[ref20] Ross C. E, Mirowsky J (2010). The Interaction of Personal and Parental Education on Health.

[ref21] The health policy document in the Islamic Republic of Iran (2015). www.Khamenei.ir.

[ref22] Tsouroufli M, Payne H (2008). medical trainers, modernizing medical careers (MMC) and the European time directive (EWTD):tensions and challenges in a changing medical education context. BMC Med Educ.

[ref23] Yasuharu T, Eiji G, Junji O, Joshua J, Fumio O, Haruo O, Osamu Takahashi T. F (2010). Undergraduate educational environment, perceived preparedness for postgraduate clinical training, and pass rate on the National Medical Licensure Examination in Japan. BMC Med Educ.

[ref24] Zhang Y, Wildemuth B. M, B Wildemuth (2009). Qualitative analysis of content. Applications of Social Research Methods to Questions in Information and Library.

